# Neuronal Inputs and Outputs of Aging and Longevity

**DOI:** 10.3389/fgene.2013.00071

**Published:** 2013-05-06

**Authors:** Joy Alcedo, Thomas Flatt, Elena G. Pasyukova

**Affiliations:** ^1^Friedrich Miescher Institute for Biomedical ResearchBasel, Switzerland; ^2^Department of Biological Sciences, Wayne State UniversityDetroit, MI, USA; ^3^Institut für Populationsgenetik, Vetmeduni ViennaVienna, Austria; ^4^Wissenschaftskolleg zu Berlin, Institute for Advanced StudyBerlin, Germany; ^5^Institute of Molecular Genetics, Russian Academy of SciencesMoscow, Russia

**Keywords:** aging, longevity, homeostasis, brain, nervous system, neuroendocrine system

## Abstract

An animal’s survival strongly depends on its ability to maintain homeostasis in response to the changing quality of its external and internal environment. This is achieved through intracellular and intercellular communication within and among different tissues. One of the organ systems that plays a major role in this communication and the maintenance of homeostasis is the nervous system. Here we highlight different aspects of the neuronal inputs and outputs of pathways that affect aging and longevity. Accordingly, we discuss how sensory inputs influence homeostasis and lifespan through the modulation of different types of neuronal signals, which reflects the complexity of the environmental cues that affect physiology. We also describe feedback, compensatory, and feed-forward mechanisms in these longevity-modulating pathways that are necessary for homeostasis. Finally, we consider the temporal requirements for these neuronal processes and the potential role of natural genetic variation in shaping the neurobiology of aging.

## Introduction

The study of aging is the study of an open system, where tissues and organs within the whole animal regularly exchange information not only with each other but also with their external environment during the course of the animal’s lifespan. These exchanges in information allow the animal to maintain a stable internal environment, known as homeostasis, which is necessary for survival amid the constant flux in the animal’s external environment. An important node within this flow of information is the nervous system, which serves as an interface between the animal’s external and internal environments. Not surprisingly, neuronal signaling activities and their regulation have a major influence on the animal’s survival and aging process. Here we address the role of the nervous system in maintaining homeostasis and its consequent impact on longevity and aging.

## Signaling Networks: Intracellular, Intercellular, and Interorgan Communication in Homeostatic Maintenance – the Influence on Lifespan

**Figure 1:**
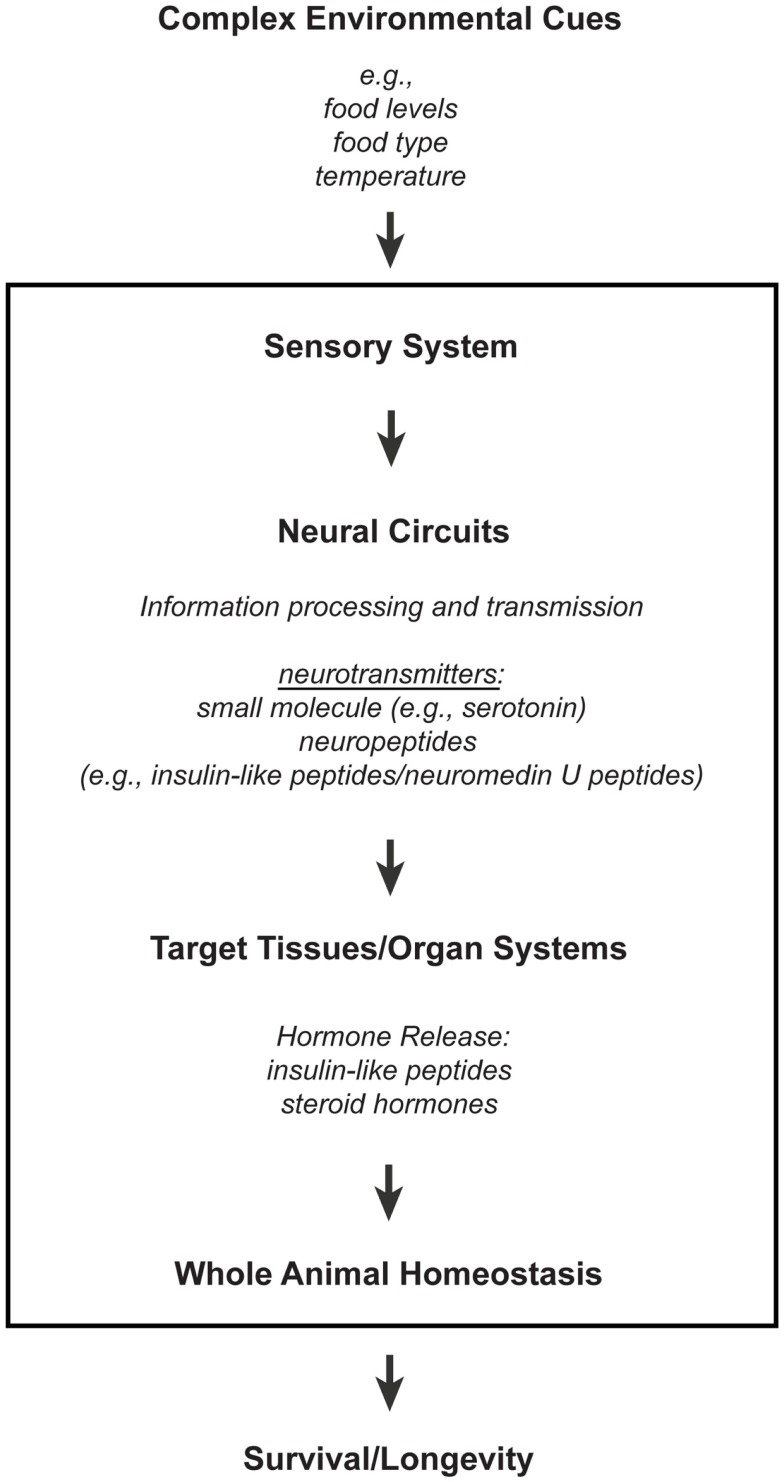
**A model for how the nervous system processes environmental information through neuronal and non-neuronal circuits to maintain homeostasis for optimal survival**. Information processing of environmental inputs at the neuronal level will involve the function of (1) small molecule neurotransmitters and neuropeptides, such as insulin-like peptides, (2) stress-sensing pathways, and (3) mitochondria-associated signals. These neuronal signaling outputs will in turn target other tissues to regulate the production of secondary signals, like hormones, and thus promote homeostasis and longevity. Insulin-like peptides can function either as short-range peptide neurotransmitters (Chen et al., 2013) or as peptide hormones.

The nervous system is a network of specialized cells that relay information between different organ systems and the environment. Sensory neurons perceive environmental cues, whose information are transmitted to non-neuronal tissues either directly or indirectly via neural circuits that consist of interneurons and/or other types of neurons, like motor neurons. These intercellular and interorgan communications involve different types of signaling molecules that range from small molecule neurotransmitters to neuropeptides and hormones (Figure 1; reviewed in Alcedo et al., 2010). Indeed, consistent with the findings that the nervous system affects longevity, the processing of environmental information by sensory neurons and the corresponding neural circuitries can modulate hormonal secretions that maintain homeostasis (Figure 1; reviewed in Alcedo et al., 2010).

### Sensory influence on homeostasis and lifespan

Sensory perception can alter a number of physiological processes, from circadian clocks (Wurtman et al., 1963, 1964; la Fleur et al., 2001; Challet et al., 2003; Ha et al., 2006), developmental plasticity (Bargmann and Horvitz, 1991; Schackwitz et al., 1996) and metabolism (Zafra et al., 2006; Greer et al., 2008) to reproduction (Yoon et al., 2005), and stress responses (Prahlad et al., 2008). Similarly, sensory neurons have been found to affect lifespan in the nematode worm *C. elegans* (Apfeld and Kenyon, 1999; Alcedo and Kenyon, 2004; Bishop and Guarente, 2007; Lee and Kenyon, 2009) and in the fruit fly *Drosophila* (Libert et al., 2007; Poon et al., 2010). This influence on lifespan involves positive or negative inputs from gustatory, olfactory, and thermosensory neurons that can modulate the activities of different peptide or steroid hormones (Apfeld and Kenyon, 1999; Alcedo and Kenyon, 2004; Libert et al., 2007; Lee and Kenyon, 2009), which would in turn presumably affect different homeostatic mechanisms (reviewed in Fielenbach and Antebi, 2008; Kenyon, 2010). The above studies demonstrating the sensory influence on *C. elegans* and *Drosophila* lifespan have been reviewed in greater detail by Jeong et al. (2012), as part of this Research Topic.

The nature of some of these neurons suggests that some of the cues that affect lifespan are food-derived, which agrees with the observation that some olfactory inputs are involved in the lifespan effects of restricting food intake levels (Libert et al., 2007), a phenomenon that is commonly known as calorie restriction (Klass, 1977; Weindruch and Walford, 1988). However, the longevity-promoting effects of food-level restriction are linked to changes in feeding rates, delayed development, and decreased reproduction (Klass, 1977; Weindruch and Walford, 1988). In contrast, the sensory influence on lifespan does not always correlate with the sensory effects on feeding behaviors, development, and reproduction (Apfeld and Kenyon, 1999; Alcedo and Kenyon, 2004; Poon et al., 2010), which suggests that the sensory system will affect lifespan through more than one mechanism. This would be expected since different types of sensory neurons can perceive a wide variety of environmental cues, ranging from temperature (Lee and Kenyon, 2009; Xiao et al., 2013) or the inherent complexity of food sources (Libert et al., 2007; Maier et al., 2010; Poon et al., 2010) to other types of cues, many of which can potentially alter organismal homeostasis and affect lifespan.

Recently, the sensory system has been shown to promote another form of dietary influence on lifespan – dependence on food-type/composition, which is distinct from the lifespan effects of food-level restriction (Maier et al., 2010). This is consistent with the previous observation that only a subset of gustatory and olfactory neurons affects lifespan in a given environment (Alcedo and Kenyon, 2004), i.e., the presence of a specific set of lifespan-influencing cues in some food sources will only be detected by a specific set of sensory neurons. Indeed, this is supported by the recent identification of a monocarboxylate-like transporter (MCT-1) that mediates the lifespan effects of only certain sensory neurons, suggesting that MCT-1 will transport some, but not all, small metabolites (Gaglia et al., 2012).

The sensory influence on lifespan via food-type recognition has also been shown to involve the activities of specific neuropeptide signaling pathways under certain environmental conditions (Maier et al., 2010). For example, a neuropeptide neuromedin U pathway processes food-type information that alters *C. elegans* lifespan, independent of food intake levels (Maier et al., 2010). Considering that many species have a large repertoire of neuropeptide ligands and receptors, many of which are expressed in the nervous system (Bargmann, 1998; Strand, 1999), these neuropeptide signaling pathways could presumably process distinct sets of sensory information into physiological responses that would optimize survival.

### Modulation of lifespan and aging by neuronal insulin/IGF signaling

The sensory influence on lifespan can be mediated by insulin/insulin-like peptides (ILPs) and their corresponding signaling pathway(s), IIS (Apfeld and Kenyon, 1999; Alcedo and Kenyon, 2004), which are also known to play a central role in regulating various aspects of growth, development, metabolism, and reproduction. Indeed, among the molecular pathways known to affect longevity, IIS is probably the best-known, and perhaps the most important, mainly due to its major, evolutionarily conserved effects on lifespan in various model organisms, from invertebrates to mammals (reviewed in Tatar et al., 2003; Taguchi and White, 2008; Partridge et al., 2011). Here we provide a brief overview of recent studies suggesting that, among the many tissues affected by this endocrine pathway, IIS action in the central nervous system (CNS) is of special importance for modulating aging and longevity (reviewed in Broughton and Partridge, 2009).

IIS in the CNS has essentially two roles in aging. On the one hand, it can have local, neuroprotective effects in the CNS itself, for example, by promoting neuronal survival under neurodegenerative conditions (Chrysis et al., 2001; Schubert et al., 2004; Plum et al., 2005; Bateman and McNeill, 2006). On the other hand, in response to environmental cues, some of which could be food-derived, CNS-acting factors could regulate the production and release of ILPs, which in turn systemically act to influence whole-organismal aging. Here we focus on such CNS-mediated, lifespan-promoting effects of reduced IIS in worms, flies, and mice (reviewed in Tatar et al., 2003; Fielenbach and Antebi, 2008; Alcedo et al., 2010).

The worm *C*. *elegans* has 40 genes that are predicted to encode ILPs, many of which are expressed in sensory neurons and interneurons and can function as ligands for the insulin receptor ortholog DAF-2 (Pierce et al., 2001; Li et al., 2003; Cornils et al., 2011). Consistent with the notion that sensory neurons produce and release ILPs that regulate lifespan by influencing IIS in remote tissues, mutations that cause defects in ciliated sensory neurons or targeted ablation of gustatory and olfactory neurons extend lifespan in a manner that is fully or partially dependent on DAF-16/FOXO, a forkhead transcription factor downstream of IIS that becomes activated when IIS is reduced (Apfeld and Kenyon, 1999; Alcedo and Kenyon, 2004; Shen et al., 2010). The central role of the CNS in the IIS modulation of longevity is further underscored by the fact that the extended lifespan due to mutations in *daf-2* and *age-1/PI-3K*, a central kinase downstream of DAF-2, can be largely or fully rescued, when wild-type *daf-2* or *age-1* is expressed in the neurons of the corresponding mutants (Wolkow et al., 2000; Iser et al., 2007). In contrast, neuronal activity of DAF-16/FOXO seems to be less important for lifespan extension in animals with impaired IIS (Libina et al., 2003; Iser et al., 2007; also, see below). However, expression of the microRNA *mir-71* in the nervous system mediates the lifespan extension in germline-ablated worms in a fashion that depends upon intestinal DAF-16 activity, revealing a complex signaling interaction between the CNS, the intestine, and the gonad in IIS-mediated lifespan regulation (Boulias and Horvitz, 2012).

Work in the fruit fly *Drosophila melanogaster* reveals remarkable parallels to these observations in worms. In the adult fly, three out of seven distinct ILPs are produced in specialized median neurosecretory cells (also called insulin-producing cells, IPCs) in the pars intercerebralis of the CNS (Rulifson et al., 2002; Grönke et al., 2010), and ablation of the IPCs significantly extends lifespan (Wessells et al., 2004; Broughton et al., 2005; Haselton et al., 2010), presumably due to reduced levels of ILP2, ILP3, and ILP5 (Broughton et al., 2008; Grönke et al., 2010). Consistent with these observations, several factors that regulate the production and/or release of ILPs affect IIS and lifespan. These factors include the metabotropic GABA receptors or uncoupling proteins (UCPs) expressed in the IPCs (Fridell et al., 2009; Humphrey et al., 2009; Enell et al., 2010) and short neuropeptide F (sNPF) expressed in the CNS (Lee et al., 2008, 2009). In addition, downregulation of p53 in the IPCs extends lifespan by reducing ILP levels and inhibiting PI-3K activity in peripheral tissues (Bauer et al., 2007). Similarly, the stress-responsive Jun kinase (JNK) in the IPCs promotes longevity by downregulating ILP2 through activation of FOXO (Wang et al., 2005). In contrast, and similar to the above-mentioned findings in *C. elegans*, activation of FOXO in the CNS, either pan-neuronally, in the neurolemma or in glial cells, is not sufficient to extend lifespan, whereas its downregulation in head fat body tissues promotes longevity (Hwangbo et al., 2004).

In mammals, the CNS also seems to play an important role in regulating the production and release of insulin-like hormones, although the bulk of insulin or IGF-1 is produced outside the brain. For example, mice with certain mutations affecting the so-called hypothalamic-pituitary-somatotropic growth hormone (GH-IIS) axis, known to regulate the release of insulin/insulin-like hormones, are long-lived, presumably due to downregulation of IIS (reviewed in Tatar et al., 2003; Holzenberger et al., 2004; Berryman et al., 2008). More direct evidence for a role of IIS in affecting mammalian lifespan via the nervous system comes from studies with transgenic or mutant mice with impaired IIS. Mice with a brain-specific deletion of the *insulin receptor substrate-2* (*Irs2*) locus are 14% longer lived than control mice, despite being hyperinsulinemic, obese, and insulin-resistant (Taguchi et al., 2007). Similarly, partial genetic inactivation of the *IGF-1 receptor* (*IGF-1R*) gene in the embryonic mouse brain inhibits GH and IGF-1 signaling after birth, which leads to growth retardation, small adult size, metabolic changes, and prolonged mean lifespan (Kappeler et al., 2008).

While much future work remains to be done for a detailed understanding of the underlying regulatory mechanisms, the available studies in worms, flies, and mice to date clearly show that neuroendocrine processes in the CNS are critically important for modulating the lifespan effects of IIS.

### The effects of neuronal stress-sensing pathways on lifespan and aging

The nervous system not only perceives a variety of environmental stressors but also integrates these information, which are then converted into appropriate physiological and behavioral adaptive responses. Below we discuss two such examples and their possible consequent effects on lifespan.

Exposure to acute stress, like heat, heavy metals, or toxins, can lead to proteotoxicity, as a result of protein misfolding within the animal (reviewed in Åkerfelt et al., 2010). To survive such insults, the animal activates its heat shock response, which is mediated by the heat shock transcription factor 1 (HSF-1; (Hsu et al., 2003; Morley and Morimoto, 2004; Cohen et al., 2006). For example, Kourtis et al. (2012) have shown that HSF-1 is required to protect the animal against cytotoxicity that is induced by thermal or other stresses through activation of the small heat shock protein HSP-16.1. This mechanism, which also protects against neurodegeneration, has been found to be conserved across species (Kourtis et al., 2012). Since thermosensory neurons and their associated neuronal circuitry can regulate the *C. elegans* heat shock responses non-autonomously (Prahlad et al., 2008; Prahlad and Morimoto, 2011), it is possible that the sensory regulation of the HSF-1/HSP-16.1 response is similarly conserved.

However, HSF-1 activity promotes longevity not only in the presence, but also in the absence, of acute stress (Hsu et al., 2003; Morley and Morimoto, 2004). Intriguingly, protein misfolding, whether it is mediated (Morley et al., 2002; van Ham et al., 2010) or not (David et al., 2010) by polyglutamine repeats, increases with age. This suggests that protein aggregation is inherent with age and is not restricted to a subset of proteins that have been implicated in diseases like neurodegeneration (David et al., 2010). Hence, given the role of HSF-1 in promoting protein disaggregation (Cohen et al., 2006), it is not surprising that HSF-1 activity in multiple tissues affects lifespan even in the absence of acute stress (Hsu et al., 2003; Morley and Morimoto, 2004).

Animals also employ different sensors for different types of gases that are required and/or affect important physiological processes. Some examples are the mechanisms through which animals perceive oxygen levels within their environment. For example, environmental oxygen is sensed by specific soluble guanylyl cyclases (sGCs) in specific sensory neurons in *C. elegans* and *Drosophila* (Cheung et al., 2005; Chang et al., 2006; Rogers et al., 2006; Vermehren-Schmaedick et al., 2010). These sGCs regulate the aerotactic behaviors of the animals: *C. elegans* prefers 7–11% ambient oxygen and is repelled by hypoxic (<5% O_2_) and hyperoxic (>14% O_2_) environments (Cheung et al., 2005; Chang et al., 2006; Rogers et al., 2006); whereas *Drosophila* larvae prefer a more restricted range of O_2_ concentration (∼21%) (Vermehren-Schmaedick et al., 2010). Besides the sGC-expressing neurons, the avoidance of hyperoxia, i.e., in *C. elegans*, also depends on the activities of neurons that sense pain and neurons that integrate information about food availability and population density (Chang et al., 2006; Rogers et al., 2006). Thus, these different sensory neurons together allow the animals to generate rapid behavioral responses to ambient O_2_, so that they can migrate to environments with the optimal O_2_ levels necessary for their survival. At present, none of the sGCs are known to affect lifespan, unlike the receptor guanylyl cyclases for which a few have been reported to inhibit longevity (Murphy et al., 2003; Alcedo and Kenyon, 2004).

There are also many other cells that respond to O_2_, albeit more slowly, through the hypoxia-inducible transcription factor HIF-1, which modifies the activities of the above O_2_-sensing neurons and existing neural circuitries (Chang and Bargmann, 2008; Pocock and Hobert, 2010). Hypoxic activation of HIF-1 shifts the animal’s preferences to lower oxygen concentrations and eliminates the dependence on some neurons, e.g., those that integrate information about food and population density, in promoting O_2_-dependent responses (Chang and Bargmann, 2008). Interestingly, this HIF-1 effect requires that it acts coordinately in neuronal and gonadal cells (Chang and Bargmann, 2008), whose outputs are known to affect lifespan (Apfeld and Kenyon, 1999; Hsin and Kenyon, 1999; Wolkow et al., 2000; Broughton et al., 2005; Flatt et al., 2008).

Indeed, the HIF-1 pathway has been recently found to influence *C. elegans* lifespan and that these lifespan effects depend on environmental context (Chen et al., 2009; Mehta et al., 2009; Zhang et al., 2009; Lee et al., 2010; Leiser et al., 2011). Particularly, loss of *hif-1* can extend *C. elegans* lifespan at higher temperatures (25°C; Chen et al., 2009; Leiser et al., 2011) or shorten lifespan at lower temperatures (20°C; Mehta et al., 2009; Lee et al., 2010). Since O_2_ perception can be modulated by food-derived information (Chang et al., 2006; Rogers et al., 2006; Chang and Bargmann, 2008; Pocock and Hobert, 2010), these temperature-dependent effects of HIF-1 may also reflect differences in the animal’s bacterial food sources grown at 25 versus 20°C. Consistent with this idea, an interaction between HIF-1 and the food-dependent TOR pathway has been observed in affecting lifespan (Chen et al., 2009). Likewise, because O_2_-sensing is also subject to population density (Chang et al., 2006; Rogers et al., 2006), the *hif-1* lifespan effects observed by Zhang et al. (2009) might reflect the higher density of animals used in their assays. Thus, HIF-1 function nicely illustrates how environmental context and its perception can modulate the effects of a signaling pathway on lifespan.

### The role of mitochondria in brain aging and longevity

Mitochondria are among the most important cellular organelles that contribute to the aging process, mainly through respiratory chain dysfunction, changes in redox status, or by generating reactive oxygen species (ROS; Humphries et al., 2006; Mattson, 2006). It is therefore not surprising that the nervous system exhibits a highly active mitochondrial metabolism, especially because of the high energetic demands associated with processes such as ion homeostasis, neurotransmission, or the firing of action potentials.

Indeed in mammals, structural impairments in mitochondrial DNA and an age-dependent reduction in brain mitochondrial function are correlated with the age-dependent decrease in cognitive function and neuromuscular coordination (reviewed in Bishop et al., 2010; Escames et al., 2010; Chakrabarti et al., 2011; Yin et al., 2012). Similarly, mitochondrial dysfunction has been implicated in neurodegenerative diseases (reviewed in Eckert et al., 2011; Reddy and Reddy, 2011; Swerdlow, 2011; Troulinaki and Bano, 2012; Yin et al., 2012), although it remains unclear whether the functional changes seen in the healthily aging brain are distinct from the pathological processes associated with neurodegenerative diseases. The empirical evidence at hand today thus suggests that neuronal mitochondria play an important role in maintaining organismal homeostasis and in influencing aging.

**Figure 2:**
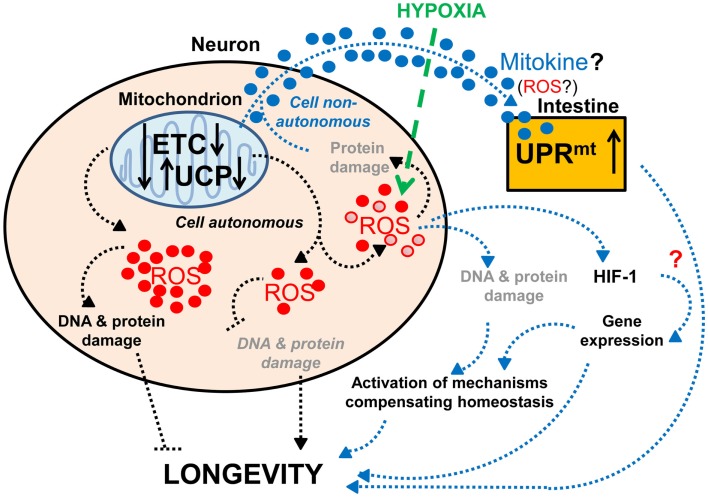
**Effects of neuronal mitochondrial UCP and the electron transport chain on longevity**. Lifespan is modulated by altered mitochondrial function in neurons: a lower level of UCP and electron transport chain (ETC) expression lengthens lifespan, whereas a higher level of UCP and ETC expression has the opposite effect on lifespan (Fridell et al., 2005; Rea et al., 2007; Copeland et al., 2009; Humphrey et al., 2009; Durieux et al., 2011). The lifespan increase observed with mild mitochondrial dysfunction may hypothetically be due to (1) a decrease in ROS production and DNA and protein damage (denoted in gray and italics) or (2) a mild increase in ROS production and DNA and protein damage (denoted in gray), which can activate compensatory mechanisms. Alternatively, mitochondria-dependent lifespan increases might also be due to other compensatory mechanisms induced by a change in the types of ROS produced (red • versus red ^). Neuronal mitochondrial dysfunction can also induce a cell non-autonomous UPR^mt^ in intestinal cells and lead to lifespan extension, via a proposed mitokine, like ROS (Durieux et al., 2011). However, intestinal UPR^mt^ response is necessary but not sufficient to promote longevity (Durieux et al., 2011). Since HIF-1 activates survival genes in response to hypoxia and a mild inhibition of mitochondrial ETC, which involves an increase in ROS levels (Lee et al., 2010), it is tempting to speculate about the possible role of HIF-1 in this process (denoted by a red “?”).

Several observations support the importance of proper neuronal mitochondrial function for lifespan and healthy aging. As mentioned previously, expression of human mitochondrial UCPs, which can uncouple mitochondrial respiration from ATP synthesis, in the neurons of adult flies extends lifespan (Fridell et al., 2005, 2009; Humphrey et al., 2009). This effect is likely to occur through reduced secretion of ILPs (Fridell et al., 2009; Humphrey et al., 2009), since the human UCP2 is known to regulate insulin secretion (Zhang et al., 2001). Interestingly, while moderate levels of neuronal UCP expression lengthen lifespan (Fridell et al., 2005; Humphrey et al., 2009), high levels have the opposite effect (Humphrey et al., 2009; Figure 2). This is reminiscent of previous studies that show a mild reduction of mitochondrial function can extend lifespan, whereas a strong functional impairment shortens lifespan (Rea et al., 2007). Therefore, hypothetically, mild mitochondrial dysfunction may cause (1) a change in levels of ROS production, e.g., a decrease that ensures preservation of DNA and protein structures or a mild increase that leads to compensatory mechanisms, or (2) a change in the types of ROS produced, which would then stimulate the expression of longevity-promoting genes. Together these data suggest that the increased lifespan associated with mild impairment of neuronal mitochondrial function (Dillin et al., 2002a; Rea and Johnson, 2003; Morrow et al., 2004; Fridell et al., 2005, 2009; Conti et al., 2006; Rea et al., 2007; Copeland et al., 2009; Humphrey et al., 2009; Lee et al., 2010; Figure 2) represents a compensatory mechanism that enables the maintenance of homeostasis.

A reduction of the function of the mitochondrial respiratory chain in the nervous system has also been shown to induce a mitochondria-specific unfolded protein response (UPR^mt^) in intestinal cells and to extend lifespan (Durieux et al., 2011; Figure 2). Interestingly, a similar impairment of mitochondrial function in muscle cells can also induce UPR^mt^, but this does not cause lifespan extension (Durieux et al., 2011), which could suggest that UPR^mt^ by itself is not sufficient for promoting longevity. On the other hand, the induction of UPR^mt^ has been found to be necessary for the long-life phenotype due to reduced mitochondrial respiration (Durieux et al., 2011). Thus, these findings suggest that mitochondrial dysfunction in neurons extends lifespan by producing an unknown signal that acts together with the UPR^mt^-inducing signal. While the nature of this additional signal remains unknown, it is tempting to speculate about the possible role of the HIF-1 pathway in this process. Indeed, HIF-1 not only modifies neuronal activities (Chang and Bargmann, 2008; Pocock and Hobert, 2010), but also promotes longevity in response to mild inhibition of mitochondrial respiration through increased ROS levels (Lee et al., 2010). Although the longevity-promoting effects of increased ROS (Lee et al., 2010) contradict a previous hypothesis that ROS would shorten lifespan through increased oxidative damage (Humphries et al., 2006; Mattson, 2006), this observation is consistent with the more recent hypothesis of mitohormesis, where higher ROS subsequently leads to increased stress resistance (Schulz et al., 2007). Alternatively, it is conceivable that certain types of ROS act as signaling molecules to activate survival pathways (Bishop et al., 2010; Lee et al., 2010; Durieux et al., 2011).

As the major source of ROS, the mitochondria are intimately involved in crosstalk among different pathways. Not surprisingly, mitochondrial activity is also regulated by major pathways that affect longevity, including the IIS, TOR, and JNK signaling pathways (reviewed in Troulinaki and Bano, 2012, as part of this Research Topic). Indeed, the ROS-mediated induction of JNK activity (Wang et al., 2005), which leads to translocation of JNK from the cytoplasm to the mitochondria, has been proposed to be of fundamental importance in the transduction of cytosolic signals to the mitochondria in the aging mammalian brain Schroeter et al., 2003; Eminel et al., 2004; Zhou et al., 2008, 2009).

Reactive oxygen species signaling itself also modulates mitochondrial homeostasis, which involves constant remodeling of this organelle, i.e., through mitochondrial fusion, fission, and autophagy (reviewed in Lemasters, 2005; Lee et al., 2012; Palikaras and Tavernarakis, 2012; Liesa and Shirihai, 2013). Such remodeling, which is tightly regulated, appears to be an adaptive response to the cell’s energy expenditure and demands (reviewed in Liesa and Shirihai, 2013). However, mitochondrial fusion and fission have also been proposed to distribute damaged organelle components across the cell’s mitochondrial network, whereas mitochondrial autophagy, known as mitophagy, removes highly damaged mitochondria (reviewed in Lemasters, 2005; Lee et al., 2012; Palikaras and Tavernarakis, 2012). Thus, an increase in ROS levels can shift the balance between fusion and fission to mitophagy (reviewed in Lemasters, 2005; Lee et al., 2012; Palikaras and Tavernarakis, 2012). Interestingly, mitophagy requires genes that have been implicated in the neurodegenerative Parkinson’s disease, i.e., the serine/threonine kinase PINK1 and the E3 ubiquitin ligase Parkin, where PINK1 senses the damaged mitochondria and recruits Parkin to induce mitophagy (Narendra et al., 2008, 2010). Thus, dysregulation of mitochondrial remodeling, including mitophagy, through excess ROS, likely contributes to the onset and progression of several age-associated neurodegenerative diseases (reviewed in Batlevi and La Spada, 2011; Palikaras and Tavernarakis, 2012).

## Feedback, Compensatory, and Feed-Forward Mechanisms in Longevity-Modulating Pathways

The studies discussed above point to the existence of major feedback mechanisms within the nervous system. Feedback loops are critically important in regulating physiology and metabolism, particularly with respect to homeostasis, and are often controlled by hormones (reviewed in Baker and Thummel, 2007; Leopold and Perrimon, 2007; Fielenbach and Antebi, 2008; Rajan and Perrimon, 2011; Hill et al., 2012). Notably, many such endocrine feedback mechanisms are thought to modulate aging and lifespan (Tatar et al., 2003; Murphy et al., 2007; Fielenbach and Antebi, 2008; Broughton and Partridge, 2009; Karpac and Jasper, 2009; Karpac et al., 2009; Tazearslan et al., 2009; Landis and Murphy, 2010), and the nervous system has been implicated in several of them (Hwangbo et al., 2004; Broughton et al., 2008; Flatt et al., 2008; Grönke et al., 2010; Alic et al., 2011; Boulias and Horvitz, 2012). Here, we focus on a few examples of feedback mechanisms that involve IIS and the nervous system.

A first example concerns the communication between adipose tissue and the brain via IIS. Hwangbo et al. (2004) found that in *D*. *melanogaster* overexpression of FOXO in the head fat body (equivalent of mammalian liver and adipose) extends lifespan and – remarkably – reduces the levels of ILP2 produced in the IPCs of the CNS, suggesting that lifespan extension is caused by FOXO-mediated negative feedback regulation of neural ILP production. This is consistent with the observation that ablation of IPCs extends lifespan (Wessells et al., 2004; Broughton et al., 2005), probably due to lowered levels of the ILP2, ILP3, and ILP5 ligands (Broughton et al., 2008; Grönke et al., 2010). Moreover, these findings are particularly interesting in view of the fact that a humoral factor produced by the fat body has been found to remotely control insulin secretion from the IPCs (Geminard et al., 2009; Tatar, 2009), yet whether this factor itself modulates lifespan remains unknown.

**Figure 3:**
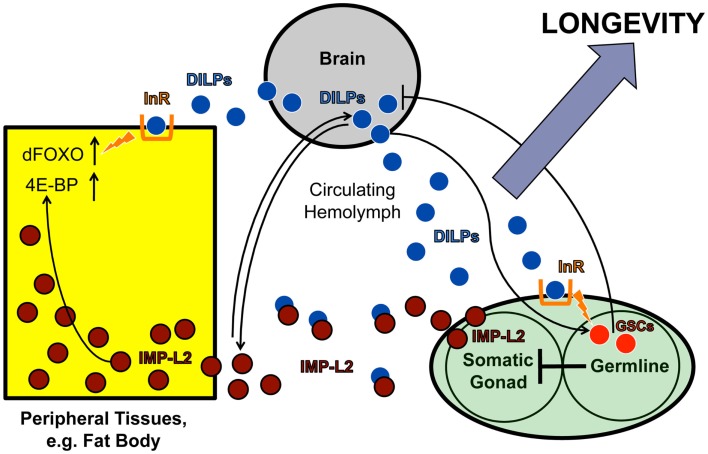
**IMP-L2-mediated endocrine feedback loop between brain and ovary**. Model of the endocrine feedback loop between brain and ovary mediated by the ILP-binding protein IMP-L2, based on findings in LaFever and Drummond-Barbosa (2005), Flatt et al. (2008), and Alic et al. (2011). ILPs produced in the brain bind to the ovarian InR and stimulate GSC proliferation. GSC proliferation likely downregulates ILP production in the IPCs since GSC ablation causes ILP transcription to increase, suggesting the existence of a negative feedback loop between the brain and ovarian tissues. This putative feedback loop might be mediated, at least in part, by the ILP-binding protein, IMP-L2, which is known to inhibit aspects of insulin signaling. Remarkably, GSC ablation results in a strong upregulation of IMP-L2. Consistent with this observation, GSC ablation and IMP-L2 overexpression cause very similar phenotypes: in both cases, flies exhibit increased lifespan, upregulation of *ilp2*, *ilp3*, and *ilp5*, and increased expression of DAF-16/FOXO targets (such as 4E-BP), although other aspects of DAF-16/FOXO activity (e.g., subcellular localization and phosphorylation status) remain unaltered. Together this suggests that the long-lifespan phenotype of GSC-ablated flies is mediated by IMP-L2, which in turn modulates insulin signaling. See text for further details.

Another example is the existence of endocrine communication between the gonad and the brain. Similar to previous findings in *C. elegans* (Hsin and Kenyon, 1999; Arantes-Oliveira et al., 2002), Flatt et al. (2008) found that ablation of germline stem cells (GSCs) extends *Drosophila* lifespan. However, despite evidence of impaired IIS in peripheral tissues, fly GSC ablation also upregulates the production of ILP2, ILP3, and ILP5 in the brain IPCs (Flatt et al., 2008). Since neurally produced ILPs are known to bind to the insulin-like receptor (InR) on GSCs to regulate GSC proliferation in the gonad (LaFever and Drummond-Barbosa, 2005; Hsu et al., 2008), it is tempting to speculate that GSCs in the gonad exert negative feedback on ILP production in the brain. Although the nature of the signal that relays this communication remains unknown, a promising candidate may be IMP-L2, an insulin-binding protein. IMP-L2, which is expressed in the germline niche, among other tissues (Terry et al., 2006), limits the availability of free ILPs by sequestering them away from the InR, thereby antagonizing systemic IIS (Honegger et al., 2008). Interestingly, this protein is upregulated in germline-less, long-lived flies exhibiting ILP overproduction (Flatt et al., 2008). Moreover, similar to the phenotypes seen in germline-less flies, the Partridge group has shown that direct upregulation of IMP-L2 itself extends lifespan and increases ILP2, ILP3, and ILP5 levels, whereas genetic deletion of the *ilp2*, *ilp3*, and *ilp5* loci decreases IMP-L2 (Grönke et al., 2010; Alic et al., 2011). Together these observations support the hypothesis that IMP-L2 is part of a gonad-brain signaling circuit that regulates neural ILP levels (Figure 3).

**Figure 4:**
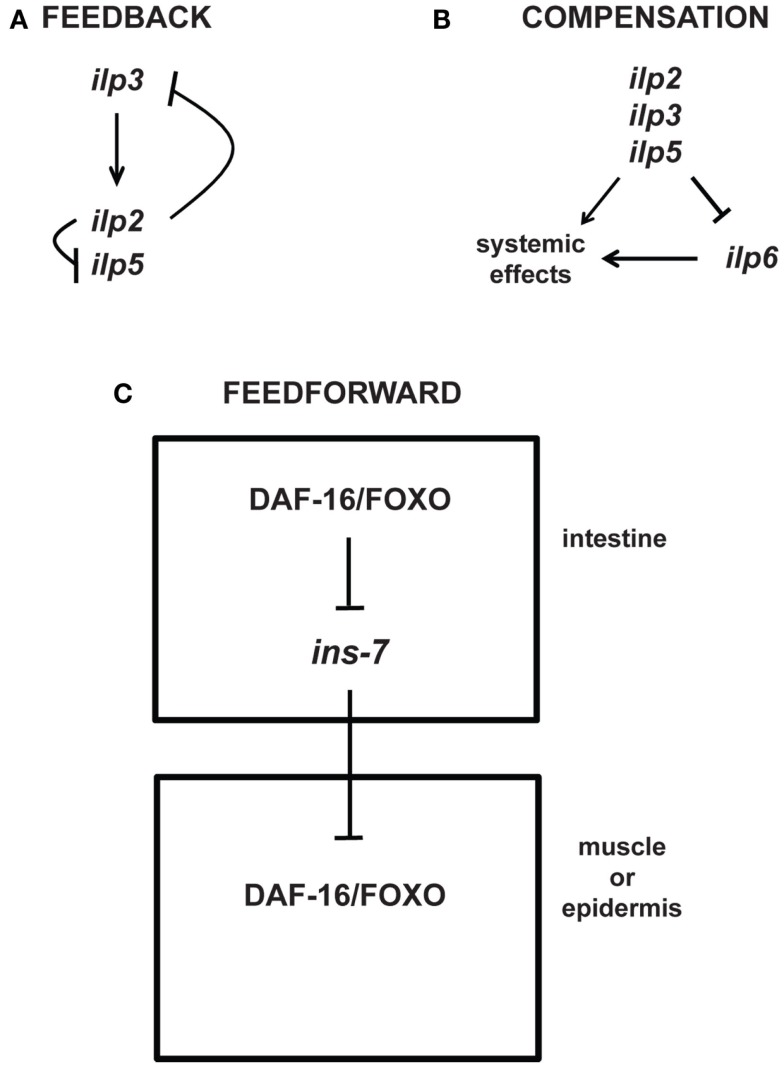
**Feedback, compensatory, and feed-forward mechanisms in the longevity-modulating insulin signaling pathway**. **(A)** Neuronally produced *Drosophila* insulin-like peptides exhibit feedback regulation among each other (Broughton et al., 2008; Grönke et al., 2010). **(B)** The systemic activities of the *Drosophila* neuronal *ilp2*, *ilp3*, and *ilp5* can be compensated by the systemic activity of the *ilp6* produced from the head fat body (Grönke et al., 2010). **(C)** *C. elegans* ILP signaling between tissues (i.e., intestine to muscle or epidermis) involves feed-forward regulation via transcriptional inhibition of the ILP *ins-7* (Murphy et al., 2007).

While the detailed consequences for physiology, and in particular for aging and longevity, are in most cases still unknown, feedback mechanisms also occur at the level of transcriptional regulation. For example, some of the seven different *Drosophila* ILPs demonstrate feedback regulation of each other (Figure 4): IPC-*expressed* *ilp3* is required for the normal expression of *ilp2* and *ilp5* in the IPCs, whereas knockdown of *ilp2* leads to upregulation of *ilp3* and *ilp5* expression in the IPCs (Broughton et al., 2008; Grönke et al., 2010). Similar feedback loops also exist for other components of IIS: *Drosophila* FOXO (dFOXO), which is activated when InR signaling is downregulated, activates the transcription of InR (Puig et al., 2003; Puig and Tjian, 2005).

Intriguingly, besides feedback loops, the genetic study of aging is also beginning to uncover other types of regulatory motifs, e.g., compensatory and feed-forward regulatory mechanisms. For instance, upregulation of the fat body-specific *ilp6* seems to compensate for the loss of the brain-specific *ilp2*, *ilp3*, and *ilp5* (Figure 4; Grönke et al., 2010). Moreover, like in *Drosophila*, *C. elegans* exhibits feed-forward regulation between ILPs (Murphy et al., 2007). Increased DAF-16/FOXO activity in a specific tissue is shown to increase the activity of DAF-16/FOXO in other tissues through feed-forward regulation that requires the inhibition of the ILP *ins-7* expression in the *C. elegans* intestine (Figure 4; Murphy et al., 2003, 2007). Thus, these studies beautifully exemplify the complexity of existing feedback, compensatory, and feed-forward mechanisms that may be relevant for modulating aging and longevity.

## Temporal Requirements of Longevity-Influencing Genes

The experimental data available today suggest that adult-expressed neuronal genes may have important effects on aging and longevity (e.g., reviewed in Broughton and Partridge, 2009). However, to what extent genes that regulate the development of the nervous system and its circuitry also influence homeostasis and longevity remains presently unclear. Interestingly, several data support the notion that early-life environmental influences might have “carry-over” effects into adulthood and might thus impact lifespan (Gavrilov and Gavrilova, 2011; Saino et al., 2012). For example, putative biomarkers of aging that affect gene activity and chromosome structure at an early age have been shown to predict life expectancy (Baeriswyl et al., 2009; Pincus and Slack, 2010; Heidinger et al., 2012). Similarly, newly emerging data from *C. elegans* show that age-related behaviors are associated with distinct transcriptomes and that the statistical analysis of these aggregate gene expression profiles can predict age and health states (Golden et al., 2008). Thus, such data tempt one to speculate that genes involved in developmental canalization (or “robustness”) might also have long-term effects on physiological homeostasis and somatic maintenance later in life. This canalization has been predicted to be a generic feature of developmental gene networks (Siegal and Bergman, 2002; Flatt, 2005).

A particularly plausible mechanism underlying these “carry-over” effects on adult lifespan is pleiotropic gene action, whereby one gene’s effect during development differs from its effect in adulthood, i.e., the same gene variant might have pleiotropic roles in affecting development versus lifespan (e.g., see Dillin et al., 2002b). On the other hand, a gene could also have different lifespan effects that depend on its temporal activity, as has been observed with the overexpression of different *p53* constructs in *Drosophila*: this can lead to different lifespan effects in females and males depending on whether expression was driven during development versus adulthood (Waskar et al., 2009). Indeed, several key lifespan modulators, like the mitochondrial electron transport chain, microRNAs, HSF-1, and FOXO, can have “carry-over” effects on adult lifespan when manipulated (e.g., overexpressed or silenced) during early larval development and/or early adulthood (Dillin et al., 2002a,b; Giannakou et al., 2007; Rea et al., 2007; Durieux et al., 2011; Pincus et al., 2011; Volovik et al., 2012). Other examples are the age-dependent expression changes in neocortical genes, which not only play a role during development but also in altered neocortical function that is observed during age-related cognitive decline and brain dysfunction (reviewed in Huffman, 2012, as part of this Research Topic).

The distinct functional roles of pleiotropic genes during development versus aging are also demonstrated by the uncoupling of their gene functions between these two processes (Chen et al., 2007; Shen et al., 2009; Thyagarajan et al., 2010). In some cases, strong loss-of-function (or null) mutations have been found to affect embryonic development in *C. elegans*, whereas weaker mutant alleles of the same gene have been shown to affect adult lifespan (Kenyon et al., 1993; Kimura et al., 1997; Gems et al., 1998; Boehm and Slack, 2005), suggesting that essential developmental genes can have deleterious effects late in life. To neutralize these late-acting deleterious effects, Liu et al. (2012) have shown that miRNA signaling is involved in specifically silencing a set of these developmental genes in adulthood, thereby restricting the pleiotropic “carry-over” effects of such genes. This is exemplified by the miRNA *miR-34*-mediated silencing of the steroid pathway gene *E74A* in *Drosophila* adults to maintain brain integrity and viability (Liu et al., 2012).

Another obvious mechanism that might play a role in “carry-over” effects on lifespan and aging are epigenetic modifications. Experiments in rodents, for instance, have shown that experiences during sensitive periods of brain development influence DNA methylation patterns, which in turn could alter gene transcription throughout life and promote specific phenotypic outcomes (Roth and Sweatt, 2011). In a similar vein, the “heterochromatin loss model of aging” posits that heterochromatin domains that are set up early in embryogenesis are gradually lost with age, which results in aberrant and age-associated gene expression patterns (Villeponteau, 1997). In support of this hypothesis, genetic manipulation of HP1 levels and JAK/STAT signaling suggests that heterochromatin formation contributes to the prevention of premature aging (Larson et al., 2012). These are intriguing preliminary observations and it will be interesting to learn more about the role of epigenetic changes in aging and lifespan in future work.

## Evolutionary Implications of Longevity-Modulating Neuronal Mechanisms

Although the classical evolutionary theory of aging posits that aging should be affected by different mechanisms in different species (Williams, 1957; Reznick, 2005), recent studies suggest that several pathways have conserved effects on longevity (reviewed in Partridge and Gems, 2002; Tatar et al., 2003; Kenyon, 2005; Partridge et al., 2005; Smith et al., 2008; Flatt and Schmidt, 2009; Fontana et al., 2010; Nakagawa et al., 2012; Wuttke et al., 2012). Whereas lifespan can vary by several orders of magnitude across different species (Finch, 1990; Stearns, 1992; Nabholz et al., 2008; Li and de Magalhães, 2011), the molecular underpinnings of longevity have so far been mainly studied in a few short-lived and genetically tractable model systems, suggesting that our current understanding of the mechanisms of aging might be biased (Deweerdt, 2012). Moreover, while many of the conserved, pleiotropic signaling pathways implicated in aging have neuronal roles, not all of these functions might directly impinge on aging. Therefore, the extent to which the neuronal mechanisms of longevity are evolutionarily conserved remains largely unclear.

A recent study directly comparing gene expression profiles during aging in mouse, rhesus macaque and human brains indicates that only a small subset of the age-dependent expression changes might be conserved (Loerch et al., 2008). These few genes include the neuroprotective gene apolipoprotein D (APOD), which is robustly upregulated with age in all three species and whose two *Drosophila* homologs are known to affect lifespan (Ruiz et al., 2011). Another example is the calcium/calmodulin-dependent protein kinase IV (CAMK4), which has been shown to regulate synaptic plasticity (Ho et al., 2000) and is downregulated with age in all three species (Loerch et al., 2008). In contrast, most genes did not show a consistent age-dependent pattern across species, leading the authors to conclude that humans and rhesus macaques have greatly diverged from mice as demonstrated by a dramatic increase in age-dependent repression of human and macaque neuronal genes (Loerch et al., 2008). While these results indicate that the neuronal mechanisms of aging and longevity might not be highly conserved among different taxa, a study by Fonseca et al. (2005) provides a remarkable counter-example. Across a range of terrestrial, freshwater, marine, tropical, and temperate arthropods, whose lifespans vary by three orders of magnitude, the neuronal deposition of lipofuscin, a lipid-protein aggregate, is highly correlated with lifespan. This suggests that age-dependent damage accumulation in the brain might be the primary driver of senescence (Fonseca et al., 2005).

Similarly, at the microevolutionary or intraspecies level, it is still unclear whether natural variation in lifespan is based on allelic variation within the same genes and pathways that have already been previously found to affect longevity in laboratory studies of mutant or transgenic model organisms (Flatt, 2004; Paaby and Schmidt, 2008; Flatt and Schmidt, 2009). On the one hand, some studies have failed to confirm the lifespan effects of natural variants of candidate longevity genes (Geiger-Thornsberry and Mackay, 2004). On the other hand, there is increasing evidence that genetic variation in candidate longevity genes might indeed contribute to variation in lifespan, as well as life history traits, in natural populations (Schmidt et al., 2000; Paaby and Schmidt, 2008; Suh et al., 2008; Paaby et al., 2010; Rose et al., 2010, 2011; Pijpe et al., 2011; Luisi et al., 2012).

A particularly striking example of such a variant is the gene FOXO3A, a human ortholog of *Drosophila* FOXO and *C. elegans* DAF-16. Several independent studies of natural polymorphisms in FOXO3A in Japanese, German, French, Italian, and Han Chinese populations have found that specific variants in this gene are associated with exceptional longevity among human centenarians (Willcox et al., 2008; Anselmi et al., 2009; Flachsbart et al., 2009; Li et al., 2009; Pawlikowska et al., 2009; Soerensen et al., 2010; Zeng et al., 2010). Although one cannot rule out a certain level of ascertainment bias, these results suggest that FOXO not only plays a functional role in regulating lifespan in laboratory model organisms, but that naturally occurring alleles can also have measurable effects on lifespan. Similar associations between natural polymorphisms and human longevity have been identified for IGF-1R (Suh et al., 2008). Likewise, evidence from *Drosophila* indicates that natural alleles in the InR locus do affect life history traits that are closely linked to longevity (Paaby et al., 2010).

Finally, a similar pattern appears to be emerging with regard to natural variants of genes involved in the neuronal regulation of lifespan: correlations have been found between longevity and genes that function in (1) neuronal development (Rybina and Pasyukova, 2010; Walter et al., 2011), (2) in neural circuitry (De Benedictis et al., 1998; De Luca et al., 2001, 2003; Carbone et al., 2006), or (3) in the uncoupling process in neuronal tissues (Rose et al., 2011).

## Conclusions and Perspectives

Here we have provided a review of the recent knowledge about the neuronal inputs and outputs that affect aging and longevity, mainly by focusing on the latest work in genetically tractable model organisms, such as flies, worms, and mice. Even though many details remain to be discovered, it is amply clear today that aging and longevity are profoundly influenced by neuronal activities. Indeed, given that the nervous system (especially, the neuroendocrine system) is intimately involved in regulating an animal’s physiology, e.g., its homeostasis and survival, in response to environmental changes, such a role for this organ system in the aging process is not surprising, both from a physiological and evolutionary perspective. Yet numerous difficult puzzles remain to be solved in future work. For example, with regard to IIS, we know that downregulation of this pathway can have positive effects on lifespan; however, at the same time such downregulation can severely impair neuronal survival and CNS function in old age (also, see discussion in Broughton and Partridge, 2009). Perhaps these distinct effects of IIS on animal physiology could depend on the tissue- or temporal-specific activities of the pathway. Hence, these pleiotropic effects of IIS highlight our need for a much better understanding of how, why, and when “brain aging” and “organismal aging” are exactly coupled or decoupled. More generally, understanding the developmental “carry-over” effects on adult lifespan will require us to gain further insight into the tissue-, age-, and stage-specificity of the neuronal effects on aging and longevity. Similarly, our current knowledge of the intricate interactions involved in the neuronal regulation of aging and longevity is still extremely rudimentary. For instance, not much is known about the interactions between different “longevity” pathways in the brain, or how different tissues (such as the gonad or adipose tissue) cross-talk with the CNS in the modulation of whole-organism lifespan. Thus, despite the fact that recent years have witnessed a lot of progress in this area, there are clearly very exciting times and novel discoveries ahead in the elucidation of the neuronal aspects of aging and longevity.

## Conflict of Interest Statement

The authors declare that the research was conducted in the absence of any commercial or financial relationships that could be construed as a potential conflict of interest.
